# Cross-Strain Neutralizing and Protective Monoclonal Antibodies against EEEV or WEEV

**DOI:** 10.3390/v13112231

**Published:** 2021-11-05

**Authors:** Amanda L. Phelps, Lyn M. O’Brien, David O. Ulaeto, Frederick W. Holtsberg, Grant C. Liao, Robin Douglas, M. Javad Aman, Pamela J. Glass, Crystal L. Moyer, Jane Ennis, Larry Zeitlin, Les P. Nagata, Wei-Gang Hu

**Affiliations:** 1 Defence Science and Technology Laboratory, Porton Down, Salisbury SP4 0JQ, UK; ALPHELPS@dstl.gov.uk (A.L.P.); LMOBRIEN@dstl.gov.uk (L.M.O.); DULAETO@dstl.gov.uk (D.O.U.); 2 Integrated BioTherapeutics, Inc., Rockville, MD 20850, USA; rick@integratedbiotherapeutics.com (F.W.H.); gliao@IntegratedBiotherapeutics.com (G.C.L.); robin@IntegratedBiotherapeutics.com (R.D.); javad@integratedbiotherapeutics.com (M.J.A.); 3 US Army Medical Research Institute of Infectious Diseases, Frederick, MD 21702, USA; pamela.j.glass.civ@mail.mil; 4 Mapp Biopharmaceutical, Inc., San Diego, CA 92121, USA; crystal.moyer@mappbio.com (C.L.M.); jane.ennis@mappbio.com (J.E.); larry.zeitlin@mappbio.com (L.Z.); 5 Defence Research and Development Canada, Suffield Research Centre, Medicine Hat, AB T1A 8K6, Canada; lnagata@drdc-rddc.gc.ca

**Keywords:** neutralizing antibodies (Nabs), anti-EEEV, anti-WEEV, in vitro neutralization assay, in vivo protective efficacy

## Abstract

The three encephalitic alphaviruses, namely, the Venezuelan, eastern, and western equine encephalitis viruses (VEEV, EEEV, and WEEV), are classified by the Centers for Disease Control and Prevention (CDC) as biothreat agents. Currently, no licensed medical countermeasures (MCMs) against these viruses are available for humans. Neutralizing antibodies (NAbs) are fast-acting and highly effective MCMs for use in both pre- and post-exposure settings against biothreat agents. While significant work has been done to identify anti-VEEV NAbs, less has been done to identify NAbs against EEEV and WEEV. In order to develop anti-EEEV or -WEEV NAbs, mice were immunized using complementary strategies with a variety of different EEEV or WEEV immunogens to maximize the generation of NAbs to each of these viruses. Of the hybridomas generated, three anti-EEEV and seven anti-WEEV monoclonal antibodies were identified with in vitro neutralization activity. The most potent neutralizers (two anti-EEEV NAbs and three anti-WEEV NAbs) were further evaluated for neutralization activity against additional strains of EEEV, a single strain of Madariaga virus (formerly South American EEEV), or WEEV. Of these, G1-2-H4 and G1-4-C3 neutralized all three EEEV strains and the Madariaga virus strain, whereas G8-2-H9 and 12 WA neutralized six out of eight WEEV strains. To determine the protective efficacy of these NAbs, the five most potent neutralizers were evaluated in respective mouse aerosol challenge models. All five NAbs demonstrated various levels of protection when administered at doses of 2.5 mg/kg or 10 mg/kg 24 h before the respective virus exposure via the aerosol route. Of these, anti-EEEV NAb G1-4-C3 and anti-WEEV NAb 8C2 provided 100% protection at both doses and all surviving mice were free of clinical signs throughout the study. Additionally, no virus was detected in the brain 14 days post virus exposure. Taken together, efficacious NAbs were developed that demonstrate the potential for the development of cross-strain antibody-based MCMs against EEEV and WEEV infections.

## 1. Introduction

The three encephalitic alphaviruses, namely, the Venezuelan, eastern, and western equine encephalitis viruses (VEEV, EEEV, and WEEV), belong to the Alphavirus genus of the family Togaviridae [1]. All three encephalitic alphaviruses share a number of sequence, structural, and functional similarities, including a positive-sense and single-stranded RNA genome with two polyprotein gene clusters, namely, nonstructural and structural [2]. The nonstructural proteins are translated directly from the 5′ two-thirds of the genomic RNA. A subgenomic positive-stranded RNA (the 26S RNA) is identical to the 3′ one-third of the genome and serves as the translational template for the structural proteins, capsid (C), E3, E2, 6K, and E1 [3]. Three of these proteins, C, E1, and E2 are found on all mature encephalitic alphavirus virions, while the E3 protein has only been positively identified in VEEV capsids to date [4]. The C encapsidates the viral genome and lies beneath the viral lipid bilayer. E1 and E2 project from the virus envelope as trimeric spikes of E1/E2 heterodimers. The E1 protein is responsible for membrane fusion, while E2, the receptor-binding protein, is believed to be the major protective antigen [5,6]. EEEV formerly encompassed North American and South American strains, with the North American strains being much more virulent than their South American counterparts. The South American strains are now classified as a separate species, namely, the Madariaga virus, which has an approximately 23% difference in nucleotide sequence from EEEV [7].

In nature, these viruses primarily circulate through animal populations and infect humans via bites from mosquito carriers that have fed on infected animals. Human infection typically results in an acute and highly incapacitating disease that is characterized by severe symptoms that are similar to influenza. However, severe or fatal encephalitis can result from these viruses crossing the cerebral vascular endothelium or the olfactory epithelium [8]. In 2019, the northern USA provinces experienced the worst outbreak of EEEV since monitoring of the disease began 15 years ago and a total of 38 cases, including 12 deaths, were reported [9].

Accidental laboratory infections [1] and experimental studies in animals [10] with these three alphaviruses have demonstrated that they are highly infectious via the aerosol route. Furthermore, alphavirus infections via the aerosol route develop much faster, displaying higher morbidity compared with the natural (mosquito bite) route, likely because the aerosol route allows for more of the virus to contact olfactory neurons, thus expediting viral invasion of the brain [11]. In addition, high titers of these alphaviruses are easily obtained in cell culture and are relatively stable (either liquid or dry) in the environment. As such, VEEV, EEEV, and WEEV are classified by the Centers for Disease Control and Prevention (CDC) as biothreat agents [12]. Indeed, VEEV was weaponized and aerosolized as an incapacitating agent by the pre-1992 Soviet Union and pre-1969 United States biological warfare programs [13]. Currently, no licensed vaccines or therapeutics are available for any of these three encephalitic alphaviruses for the protection or treatment of humans [14].

Antibodies, which are naturally produced in the body as part of the immune response to infectious agents, can also be used in the form of polyclonal serum/plasma preparations or recombinantly manufactured neutralizing antibodies (NAbs) to passively immunize patients to prevent or treat infectious diseases [15,16,17,18]. NAbs can confer immediate and consistent protection against infectious agents when administered, regardless of the recipient’s immune status. The use of NAbs in an infectious disease emergency has been demonstrated in the 2010 and 2012 outbreaks of Hendra virus in Australia [19], and in the unprecedented 2013–2016 outbreak of the Ebola virus in West Africa [20], highlighting that NAbs can play a critical role in the management of a deadly infectious disease crisis. NAbs that are licensed by regulatory agencies are available for prophylaxis of respiratory syncytial virus and therapeutically to treat anthrax [21,22], demonstrating their utility as effective medical countermeasures (MCMs) against infectious agents [23,24].

While considerable progress has been made in the development of potential therapeutic anti-VEEV NAbs [25,26,27], the development of anti-EEEV and anti-WEEV NAbs has not been significantly explored. To date, there have been only two reports of anti-WEEV NAbs [28,29], and two reports of anti-EEEV NAbs [30,31]. To address this, we set out to generate and identify NAbs against these viruses. Two different approaches were used to generate anti-EEEV and anti-WEEV neutralizing hybridoma clones, yielding a total of three anti-EEEV and seven anti-WEEV NAbs. Among them, two anti-EEEV NAbs, namely, G1-2-H4 and G1-4-C3, and two anti-WEEV NAbs, namely, G8-2-H9 and 12 WA, were shown to possess cross-strain neutralizing activity against multiple strains of EEEV and WEEV, respectively. In addition, in an in vivo pre-exposure prophylaxis setting, all five NAbs that were tested demonstrated a level of protection against EEEV or WEEV aerosol infection. 

## 2. Materials and Methods

### 2.1. Reagents, Cells, and Viruses 

High-glucose Dulbecco’s minimal essential medium (DMEM), Leibovitz (L-15) medium, phosphate-buffered saline (PBS), non-fat dry milk, fetal bovine serum (FBS), Synth-a-Freeze defined protein-free cryopreservation medium, Tween-20, SDS-polyacrylaminde gels, StartingBlock (PBS) blocking buffer, polyvinylidene difluoride (PVDF) membrane, agarose, penicillin, streptomycin, Melon Gel purification kits, Nunc cryovials, and Invitrogen cloning kits were purchased from ThermoFisher Scientific (Loughborough, UK; Waltham, MA, USA; Ottawa, ON, Canada) or Sigma Aldrich (Poole, UK). TiterMax Gold adjuvant was purchased from Cedarlane (Burlington, ON, Canada). Clonacell-HY Kit was from Stem Cell Technologies (Vancouver, BC, Canada). The AdEasy system was purchased from Qbiogene (Carlsbad, CA, USA). Restriction enzymes were from New England Biolabs (Mississauga, ON, Canada). Cell culture flasks and plates were from VWR (Mississauga, ON, Canada). Goat anti-mouse IgG horseradish peroxidase (HRP)-conjugated antibody and 2,2′-Azino-di(3-ethylbenzthiazoline-6-sulfonate) (ABTS) substrate were from KPL (Gaithersburg, MD, USA). Isoflurane was obtained from Baxter (Mississauga, ON, Canada). Alkaline phosphate substrate and goat anti-mouse alkaline phosphate conjugated antibody were from Bio-Rad Laboratories (Hercules, CA, USA). A non-specific murine IgG monoclonal antibody (MAb) and dimethyl sulfoxide (DMSO) were obtained from Sigma Aldrich (Poole, UK).

Vero (ATCC CCL-81) cells, Sp 2/0 mouse myeloma cells, and HEK 293 cells were from the American Type Culture Collection (ATCC, Manassas, VA, USA), and the European Collection of Animal Cell Cultures (Salisbury, UK). 

EEEV strains PE6, FL93-969, and Williams; Madariaga virus (435731); and WEEV strains B11 and CBA-87 were kindly provided by Dr. George Ludwig, United States Army Medical Research Institute for Infectious Diseases (USAMRIID), Frederick, MD, USA. WEEV strain 71V-1658 was kindly provided by Dr. Nick Karabatsos, CDC, Fort Collins, CO, USA; WEEV strains Fleming and California were purchased from ATCC; WEEV strains McMillan, Mn548, and Mn520 were kindly provided by Drs. Mike Drebot and Harvey Artsorb, National Microbiology Laboratory, Winnipeg, MN, Canada. 

### 2.2. Cell and Virus Maintenance

Hybridoma cell lines were maintained in DMEM supplemented with 10% FBS. HEK 293 cells were grown in DMEM supplemented with 5% FBS. Vero cells were grown in DMEM supplemented with 5% FBS, or DMEM supplemented with 10% FBS, 2 mM l-glutamine, 50 IU/mL penicillin, and 50 µg/mL streptomycin. All cell cultures were at 37 °C in a 5% CO_2_ humidified atmosphere, except for the virus culture in L15 medium, which had no added CO_2_. For cryopreservation, cell lines were resuspended in Synth-a-Freeze defined protein-free cryopreservation medium, or FBS supplemented with 20% (*v/v*) DMSO, and 1 mL aliquots were transferred to cryovials and stored in liquid nitrogen.

Experiments with live viruses were carried out at two establishments: the Defence Science and Technology Laboratory (Dstl), UK, under UK Advisory Committee on Dangerous Pathogens, Containment Level 3 (CL3) conditions, and the Defence Research and Development Canada, Suffield Research Centre (DRDC SRC) CL3 facilities in compliance with Public Health Agency of Canada and Canadian Food Inspection Agency Guidelines. 

Virulent virus stocks were prepared at Dstl by inoculating suckling mouse pups intra-cranially with the virus and allowing them to become moribund (24 h after inoculation) before culling with an overdose of sodium pentobarbital. All animal studies were carried out in accordance with the UK Animals (Scientific Procedures) Act of 1986 and the Codes of Practice for the Housing and Care of Animals used in Scientific Procedures 1989. The work was performed under Project licence 30/3166 and was approved by the UK Home Office on 16 April 2014. The virus was harvested by extracting tissue through the dorsal cranium with a large-bore syringe needle and mixed with L-15 medium supplemented with 2% (*v/v*) FBS, 2 mM l-glutamine, 50 IU/mL penicillin, and 50 µg/mL streptomycin (virus culture medium). This was then passed through a 70 µm nylon cell strainer, clarified at 10,000 rpm for 10 min in an SW28 rotor (Beckman Coulter, UK), and stored at −80 °C. Standard plaque assays were performed in a 24-well plate format with 100 µL/well of virus inoculum applied in duplicate to Vero cell monolayers under a carboxymethyl cellulose overlay. Plates were incubated in humidified conditions at 37 °C for 3–4 days prior to fixation with 10% (*v/v*) formal saline solution and visualization of plaques was achieved using 1% crystal violet solution. The limit of detection in this assay is 10 plaque-forming units (pfu)/mL of the original sample. The use of this production method minimizes the potential for loss of virulence factors as a result of cell culture adaptation, and also provides a more representative wild-type virus population (quasispecies). 

Virus seed stocks were made at DRDC SRC via the inoculation of Vero cells with virus suspensions at a multiplicity of infection of less than 0.1. The supernates were clarified using centrifugation and stored in aliquots at −70 °C [32]. 

### 2.3. Immunogens

An encephalomyelitis vaccine for horses, consisting of formalin-inactivated EEEV and WEEV, and tetanus toxoid (Zoetis vaccine) was purchased from Zoetis Canada INC (Kirkland, QC, Canada).

An adenovirus DNA vaccine, namely, pAd-EEEV PE6, was constructed by cloning the structural proteins of EEEV strain PE6 into an adenovirus vector using the AdEasy system according to the manufacturer’s protocol. The recombinant adenoviral construct pAd-EEEV PE6 was linearized with Pac I and transfected into HEK 293 cells cultured in DMEM with 5% FBS for amplification and then the amplified adenovirus was purified via chromatography.

A plasmid DNA vaccine, namely, pVHX-6-WEEV 71V-1658, was described previously [33] and expresses the structural proteins (capsid, E3, E2, 6K, and E1) of WEEV 71V-1658.

Recombinant E2 (rE2) of EEEV V105-00210 was prepared by cloning the E2 gene into a mammalian expression plasmid for expression in HEK293 cells and bacmid expression plasmid for Baculovirus expression in SF9 cells. Each recombinant protein contained a C-terminal 8× His tag. Expressed proteins were purified using metal affinity chromatography [34].

The recombinant E1 (rE1) or rE2 antigen of WEEV Fleming was prepared by cloning the E1 or E2 gene into a pCRT7 bacterial expression vector and the C-terminal 6× His-tagged rE1 or rE2 was expressed in bacteria and purified using metal affinity chromatography [35]. 

### 2.4. The First Approach to Develop Anti-WEEV or Anti-EEEV NAbs 

The rE2 antigens of EEEV V105-00210 and WEEV Fleming were shipped to a custom antibody service provider (Precision Antibody; PA; Columbia, MD, USA) for immunization of mice and generation of hybridoma clones that were reactive to the rE2 antigen of EEEV V105-00210 or WEEV Fleming. 

A total of seven hybridoma clones that were reactive to the rE2 antigen of EEEV V105-00210 and 66 clones reactive to the rE2 antigen of WEEV Fleming were generated. 

### 2.5. The Second Approach to Develop Anti-WEEV and Anti-EEEV NAbs

**Figure 1:**
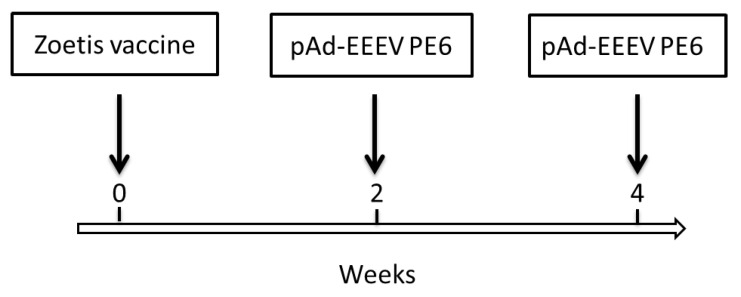
EEEV immunization scheme. Female BALB/c mice were i.m. primed with 100 µL/mouse of Zoetis vaccine at week zero and boosted with 100 µL/mouse of pAd-EEEV PE6 (100 µg) at weeks 2 and 4.

**Figure 2:**
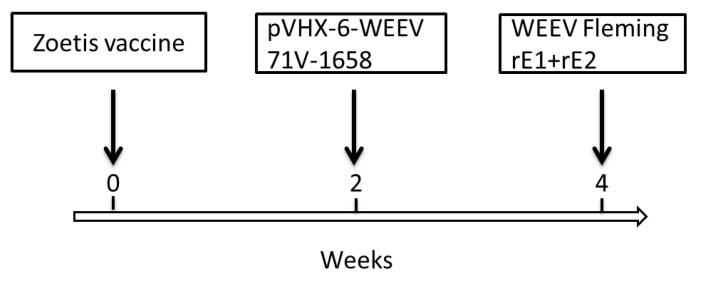
WEEV immunization scheme. Female BALB/c mice were i.m. primed with 100 µL/mouse of Zoetis vaccine at week zero and boosted with 100 µL/mouse of pVHX-6-WEEV 71V-1658 (100 µg) at week 2 and 100 µL/mouse of rE2 (100 µg) and rE1 (100 µg) of WEEV Fleming emulsified in 100 µL of TiterMax Gold adjuvant at week 4.

Female BALB/c mice (4–6 weeks old) were obtained from Charles River Canada (St Constant, QC, Canada). All mouse experiments were performed in strict accordance with the guidelines set out by the Canadian Council on Animal Care. The animal care protocol was reviewed and approved in 2013 by the Committee on the Ethics of Animal Experiments of DRDC SRC (protocol number: W1H-13-1-2-0). All efforts were made to minimize the suffering of the mice. The mice were intramuscularly (i.m.) immunized with immunogens in different formats from different strains to develop anti-EEEV or anti-WEEV Nabs, as shown in Figure 1 and Figure 2.

Three days after the last booster, five mice for each of EEEV and WEEV were sacrificed. Splenocytes were prepared and fused with myeloma cells in a standard hybridoma fusion protocol. Briefly, spleens were aseptically dissected from the immunized mice three days after the last booster, ground gently with autoclaved frosted-glass slides in DMEM, and filtered through a wire mesh screen to prepare splenocytes. Hybridomas were produced by fusing the splenocytes with Sp 2/0 myeloma cells using a Clonacell-HY Kit. After 2 weeks in a semisolid medium, five-thousand individual hybridoma clones for each virus were picked from semisolid medium and transferred to 96-well plates and cultured for 7 days in Clonacell Medium E, as previously described [36].

Hybridoma culture supernates were screened using an in vitro neutralization assay. Briefly, 20 pfu/well of EEEV PE6 or 125 pfu/well of WEEV 71V-1658 were incubated with a hybridoma culture supernate at 37 °C for 1 h. The mixtures were then added to Vero cells in 96-well plates (1 × 10^4^ cells/well). Two or three days later, cells were observed under the microscope for cytopathic effects (CPE). Hybridoma clones that suppressed CPE were expanded. NAbs were purified from the cell culture supernate using a Melon Gel purification kit according to the manufacturer’s protocol. The supernate was dialyzed for two exchanges (1 h each) in Melon Gel IgG Purification Buffer pH 7.0 and then was added to a column containing the Melon Gel resin. After a 5 min incubation with end-over-end mixing, the purified IgG was collected in the flowthrough. All purified IgG samples were stored in aliquots at −70 °C.

### 2.6. Enzyme-Linked Immunosorbent Assay (ELISA)

ELISA plates were coated with sucrose-purified WEEV CBA-87 in PBS (3 µg/well) overnight at 4 °C. Following incubation, the excess was decanted and plates were blocked with PBS containing 0.02% Tween-20 and 5% non-fat dry milk (PBSTM) for 2 h at 37 °C. Following incubation, plates were either used immediately or stored at −20 °C for up to one month. Plates were washed three times with PBS containing 0.02% Tween-20 (PBST). Samples were added to the plate in duplicate and diluted down the plate in two-fold dilutions in PBSTM with 1% heat-inactivated FBS, starting at a dilution of 1:20 to 1:80. Plates were incubated for 1–2 h at 37 °C and then washed three times with PBST. After washing, goat anti-mouse IgG HRP conjugated antibody diluted 1:50,000 in PBSTM with 1% FBS was added (100 µL/well) and then incubated for 1 h at 37 °C. After another three washes with PBST, ABTS substrate (100 µL/well) was added and incubated for approximately 10 min at 37 °C. Plates were read at 410 nm with a SpectraMax M5 plate reader (Molecular Devices, Sunnyvale, CA, USA).

### 2.7. Western Blot Analysis 

rE2 antigens of EEEV V105-00210 and WEEV Fleming, or inactivated EEEV V105-00210, were denatured under reducing conditions and run on a gradient SDS-polyacrylamide gel. The proteins were transferred to a PVDF membrane, followed by a blocking step with StartingBlock to prevent antibodies from non-specifically sticking to the membrane. The membrane was incubated with supernate from hybridoma cells secreting antibodies to the rE2 antigens, followed by a secondary alkaline phosphatase-conjugated antibody. Alkaline phosphate substrate was added to visualize antibody reactivity to the rE2 antigens or EEEV V105-00210.

### 2.8. Plaque Reduction Neutralization Test (PRNT)

Virus culture media or MAb (20 µg/mL) was mixed with an equal volume of virus suspension (400 pfu/mL) and incubated overnight at 4 °C. A total of 500 µL of each mixture was inoculated in duplicate onto Vero cells (6-well plate) and incubated for 4 days at 37 °C under a carboxymethyl cellulose overlay. After 4 days of incubation, monolayers were fixed with 10% formal saline overnight and stained with 1% crystal violet. A reduction in plaque numbers compared with positive control wells (cell culture media mixed with virus only) of ≥50% was considered to qualify as neutralization.

### 2.9. Titration of NAbs against Various Strains of EEEV or WEEV 

**Figure 3:**
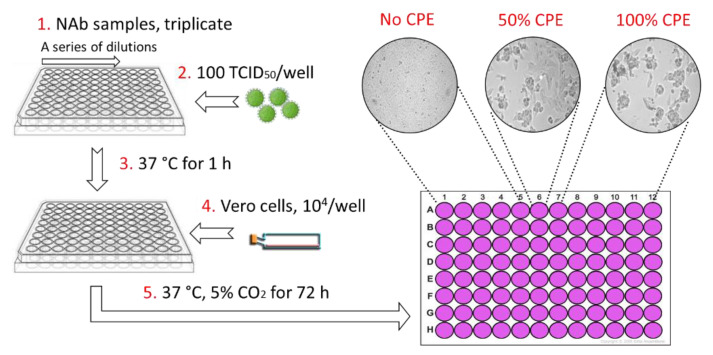
Anti-alphavirus neutralization titration assay. Serial 3-fold dilutions were made for each purified antibody in 50 µL. The virus (50 µL) was added to each well and then pre-incubated at 37 °C for 1 h. Thereafter, 1 × 10^4^ Vero cells per well were added in a volume of 50 µL. Plates were then incubated for 3 days at 37 °C with 5% CO_2_ and then examined under a microscope. The NAb titer was identified as the highest dilution that resulted in 50% inhibition of CPE caused by 100 TCID_50_.

As shown in Figure 3, the alphavirus neutralization test (ANT) was carried out in 96-well plates. Three-fold serial dilutions of each NAb were screened, starting with a maximum concentration of 100 µg/mL. The volume of each well was 50 µL. Subsequently, 100× the 50% tissue culture infective dose (TCID_50_) of the virus in 50 µL was added to each well and then pre-incubated at 37 °C for 1 h to allow for neutralization of the virus. Thereafter, 1 × 10^4^ cells/well (Vero) were added in a volume of 50 µL. Plates were then incubated at 37 °C under 5% CO_2_. After 3 days, the plates were examined microscopically. The NAb titer was identified as the highest dilution that resulted in 50% inhibition of CPE.

### 2.10. In Vivo Protective Efficacy Evaluation

In vivo studies were performed in accordance with the UK Scientific Procedures (Animals) Act 1986 and the UK Codes of Practice for the Housing and Care of Animals Used in Scientific Procedures 1989 (as well the Animal Care and Use Review Office, Fort Detrick, MD, USA). The work performed in the UK was performed under Project licence 30/3166, approved by the UK Home Office 16 April 2014. Micro-chipped female BALB/c mice, aged 7–9 weeks old (18–20 g; Charles River Laboratories, Margate, UK) were suitably housed with access to food and water ad libitum in a rigid-walled CL3 isolator. Acclimatization within the CL3 isolator was for a minimum of 5 days and all mice were weighed prior to performing any procedures. Mice were inoculated with either 2.5 or 10 mg/kg of NAb via the intraperitoneal (IP) route (100 µL/mouse) 24 h prior to aerosol exposure to a cognate virus. A non-specific murine IgG MAb was administered to a group of 5 mice at a dose of 10 mg/kg, serving as the negative control. A clinical scoring system was used to closely monitor the clinical course of infection [37] and all mice were individually weighed daily. Any mouse that was observed to have pronounced mobility issues (unable to reach food and water) was immediately culled on welfare grounds in accordance with UK Home Office Project License requirements. Mice were observed a minimum of twice daily for clinical signs of infection post-exposure by an independent technician. All culls were performed according to the UK Schedule 1 method (cervical dislocation followed by confirmation of cessation of the heartbeat). When mice succumbed to disease, the brain and lungs were excised, weighed, and stored for subsequent determination of the viral load. Additionally, all mice surviving out to 14 days post-exposure were culled and brain and lungs were similarly excised and stored. Stored organs were examined for the presence of the virus. Briefly, organs were allowed to thaw and were homogenized through a fine cell sieve (40 µm sieve, Corning Falcon cell strainer, Fisher Scientific, Loughborough, UK) into 1 mL virus culture medium. Serial dilutions were prepared from homogenates in virus culture media and titrated using a standard plaque assay.

Aerosol exposure was achieved using a 3-jet Collison nebulizer containing a minimum of 10 mL of the virus in virus culture medium without FBS and antibiotics, controlled and conditioned to 50% (±5%) relative humidity, using an AeroMP platform system (Biaera Technologies, Hagerstown, MD, USA). Mice were physically restrained in holding tubes and nose-only exposed for 10 min. A single 1 min sample of each aerosol exposure was taken using an all-glass impinger (AGI-30; Ace Glass, Vineland, NJ, USA) containing 10 mL PBS (at a flow rate of 12 L/min). The mean calculated presented challenge dose was determined using the viral titers obtained from the AGI-30 and Guyton’s formula for the respiratory volumes of laboratory animals [38].

### 2.11. Statistical Analysis

Graphs were prepared using Microsoft Excel 2010 Ink and Graphpad PRISM v8. Statistical analysis was performed using IBM SPSS (V21.0) and survival characteristics were compared using stratified and unstratified pairwise comparisons of the data using the log-rank (Mantel–Cox) test. 

## 3. Results

### 3.1. Generation and Selection of Neutralizing Hybridoma Clones

Two independent mouse immunization regimens were designed with varying immunogens to maximize the identification of novel cross-strain NAbs for EEEV and WEEV. 

First, the rE2 antigen of EEEV V105-00210 or WEEV Fleming was used to immunize the mice. As a result, 7 hybridoma clones were generated, secreting antibodies that bound to EEEV V105-00210 rE2, and 66 clones that were reactive with WEEV Fleming rE2. The seven clones that were reactive with EEEV V105-00210 rE2 were further tested via Western blotting using homologous rE2 or the whole virus as the target antigen. None of the seven MAbs bound to the rE2 or authentic E2 of EEEV in the Western blot of the denatured antigen. The 66 WEEV Fleming rE2 reactive MAbs were also tested via Western blotting, using rE2 of WEEV Fleming as the target antigen and 55 MAbs were confirmed to be capable of binding to the Western-blotted rE2 of WEEV Fleming. The 66 MAbs were also assessed using ELISA with a heterologous strain (WEEV CBA-87 as the coating antigen), where 47 MAbs were found to be able to bind to WEEV CBA-87. 

Seven EEEV rE2-reactive MAbs and the top 14 WEEV rE2-reactive MAbs based on the Western blot results were screened for their ability to neutralize the cognate viable virus. The remainder of the WEEV rE2-reactive MAbs were not further screened since their binding signals to the rE2 in the Western blot were quite low. None of the anti-EEEV MAbs effectively neutralized the EEEV PE6 infectivity for Vero cells. Meanwhile, two anti-WEEV Fleming E2 MAbs, namely, 8C2 and 8H10, neutralized the WEEV Fleming infectivity for Vero cells. 

As an alternative approach, mice were immunized with EEEV or WEEV immunogens in different formats with different strains (see Figure 1 and Figure 2). These included pAd-EEEV PE6, formalin-inactivated WEEV/EEEV viruses (Zoetis vaccine), pVHX-6-WEEV 71V-1658, and rE1/rE2 of WEEV Fleming. The hybridomas were initially screened with an in vitro neutralization assay in Vero cells to identify anti-EEEV PE6 or anti-WEEV 71V-1658 NAbs. Ultimately, three anti-EEEV and five additional anti-WEEV MAbs were identified that demonstrated neutralizing activity against EEEV PE6 or WEEV 71V-1658. 

### 3.2. NAb Titration and Evaluation against Various Strains of EEEV or WEEV

The three anti-EEEV and seven anti-WEEV NAbs were produced and purified from the hybridoma culture supernate. Anti-EEEV and anti-WEEV neutralizing activity was evaluated using ANT (Figure 3). The ANT results for anti-EEEV NAbs are summarized in Table 1. All three anti-EEEV NAbs yielded positive results, neutralizing 50% EEEV PE6 infectivity in Vero cells at concentrations ranging from 2.4 to 21.6 µg/mL. G1-2-H4 and G1-4-C3 possessed the highest efficacy and were further evaluated against two additional EEEV strains (FL93-969 and Williams) and a single strain of Madariaga virus (435731), demonstrating the neutralization of all the strains tested. 

As shown in Table 2, similar experiments with all seven anti-WEEV NAbs showed positive results, neutralizing 50% of WEEV Fleming infectivity in Vero cells at concentrations ranging from 0.03 to 18 µg/mL. Three anti-WEEV NAbs with the highest efficacy (0.03 µg/mL), namely, G8-2-H9, 12 WA, and 8C2, were evaluated against another seven WEEV strains. Two of these NAbs, namely, G8-2-H9 and 12WA, neutralized 71V-1658, CBA-87, B11, Mn548, and Mn520, but not California or McMillan. Additionally, 8C2 and 8H10 were assessed using standard PRNT; 10 µg/mL of antibodies resulted in 45% (8H10) and 100% (8C2) inhibition of plaques (mean plaque number of 87 in the untreated control wells).

### 3.3. In Vivo Protective Efficacy 

Two anti-EEEV and three anti-WEEV NAbs were down-selected for assessment in vivo to determine the protective efficacy against EEEV or WEEV aerosol challenges in a pre-exposure prophylaxis setting [37]. 

**Figure 4:**
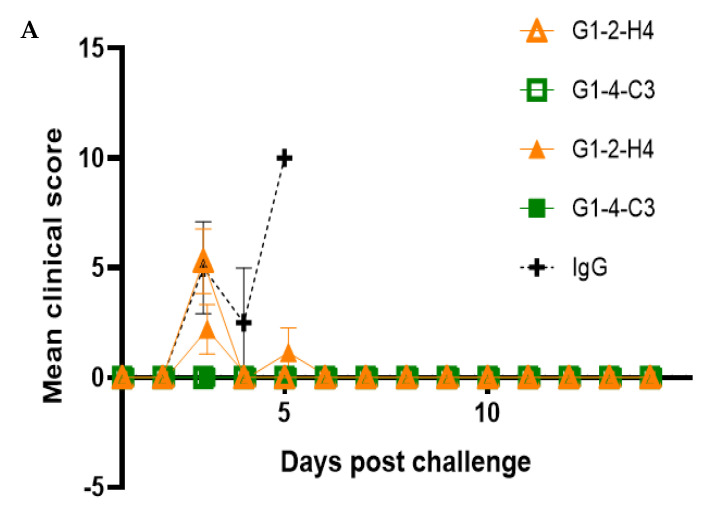

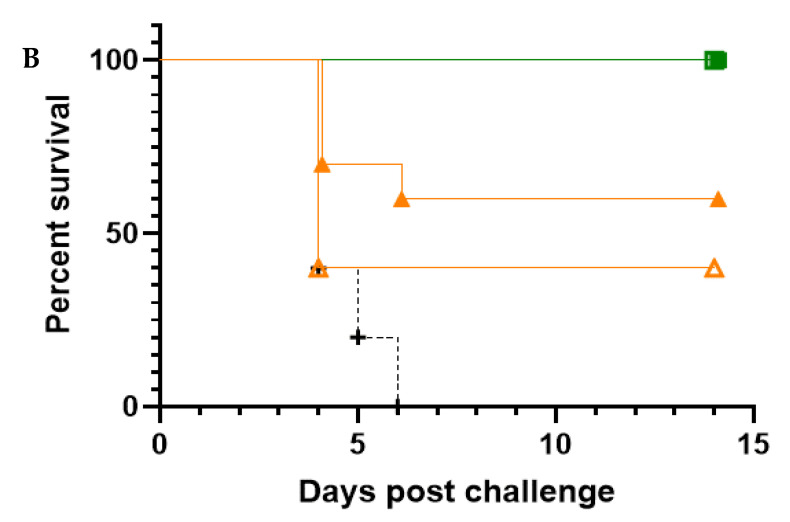
Evaluation of anti-EEEV NAb efficacy in a pre-exposure prophylaxis setting. Mean clinical score (**A**) and mortality rate (**B**) of BALB/c mice inoculated with candidate anti-EEEV NAbs at a dose of 2.5 mg/kg (empty symbols) or 10 mg/kg (filled symbols) via the IP route 24 h prior to a lethal exposure of EEEV PE6 via the aerosol route. Any deceased mice were assigned the maximum score observed in this study (*n* = 10, except for the non-specific IgG control group, where *n* = 5). Error bars indicate the standard error of the mean.

As shown in Figure 4, anti-EEEV NAb G1-2-H4 provided 40% (at a dose of 2.5 mg/kg) and 60% (at a dose of 10 mg/kg) protection, while G1-4-C3 provided 100% protection at both doses in an EEEV PE6 lethal aerosol challenge model. Surviving mice in the Nab-inoculated groups were free of clinical signs and maintained a typical weight profile throughout the study. Statistical analysis (log-rank Mantel–Cox) using pairwise comparisons identified a significant benefit in the prophylactic use of anti-EEEV NAb G1-4-C3 (*p* ≤ 0.001) at either dose when compared with the non-specific IgG control group. As mice succumbed to disease (the humane endpoint had been reached), the viral load in the brain and lungs was determined. Mice that were inoculated with either dose of G1-2-H4 that succumbed to disease (days 3–5 post aerosol exposure) had a mean of 2.17 × 10^10^ pfu/g in the brain and 2.82 × 10^4^ pfu/g in the lungs. This was comparable with viral loads that were observed in mice inoculated with non-specific IgG that succumbed to disease on days 3–5 post aerosol exposure, with a mean of 2.98 × 10^10^ pfu/g in the brain and 2.58× 10^4^ pfu/g in the lung. 

**Figure 5:**
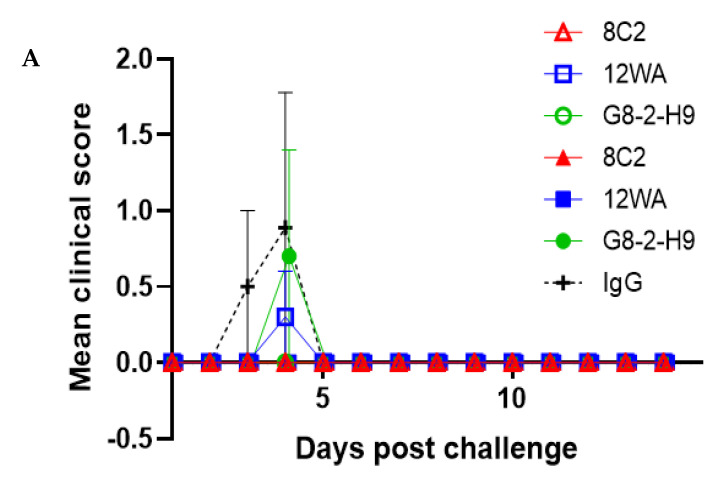

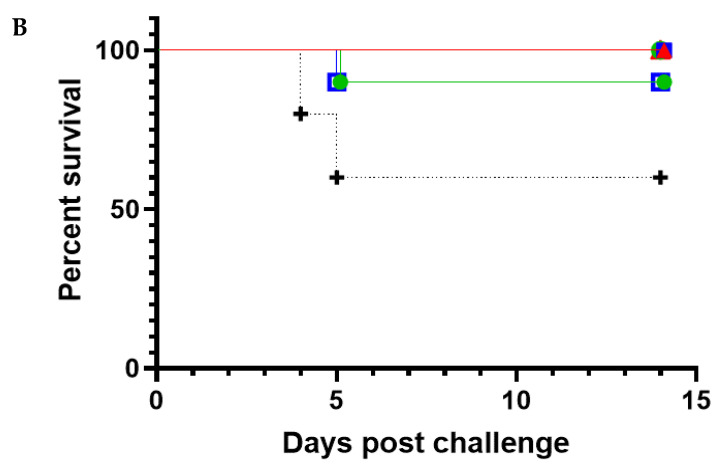
Evaluation of anti-WEEV NAb efficacy in a pre-exposure prophylaxis setting. Mean clinical score (**A**) and mortality rate (**B**) for BALB/c mice inoculated with candidate anti-WEEV NAbs at a dose of 2.5 mg/kg (empty symbols) or 10 mg/kg (filled symbols) via the IP route 24 h prior to a sublethal exposure of WEEV Fleming (7.4 MLD) via the aerosol route (*n* = 10, except for non-specific IgG control group, where *n* = 5). Error bars indicate the standard error of the mean.

In a WEEV Fleming sublethal aerosol challenge model, all three anti-WEEV Nabs, either at a dose of 10 or 2.5 mg/kg, provided up to 100% protection (Figure 5). NAb 8C2 was able to provide 100% protection when mice were inoculated with either dose. The mortality rate of control mice did not reach 100% in this study due to the lower than anticipated exposure dose of WEEV (mean of 7.4 median lethal dose (MLD)). In addition to the mortality rate, it is important to note the differences in clinical outcomes, as all NAb-treated mice were free of clinical signs (ruffled fur, hunched posture, lack of mobility, behavioral changes, and weight loss) throughout the study. Statistical analysis (log-rank Mantel–Cox) using pairwise comparisons identified a significant benefit (*p* ≤ 0.05) in the prophylactic use of these anti-WEEV NAbs when compared with the non-specific IgG control group for 8C2 at either dose, 12WA at 10 mg/kg, and G8-2-H9 at 2.5 mg/kg (*p* = 0.034). Including both doses as stratification pairwise comparisons did not identify any significant differences between the anti-WEEV NAbs in this study (*p* ≥ 0.05), highlighting the ability of all three NAbs to provide protection against aerosol exposure. During the acute phase of infection, several mice succumbed to disease (the humane endpoint had been reached) and the viral load in the brain and lungs of these animals was determined. Mice inoculated with 10 mg/kg non-specific IgG that succumbed to disease on days 3–4 post aerosol exposure achieved mean titers of 6.05 × 10^9^ pfu/g of WEEV Fleming in the brain and 1.05 × 10^2^ pfu/g in the lung. The individual mice inoculated with 2.5 mg/kg 12WA and 10 mg/kg G8-2-H9 that succumbed to disease on day 4 post aerosol exposure were also found to have high viral loads in the brain (1.3 × 10^10^ and 4.8 × 10^9^ pfu/g, respectively) and lung (3.6 × 10^7^ and 6.9 × 10^5^ pfu/g, respectively).

## 4. Discussion

The receptor-binding envelope protein E2 of alphaviruses is considered to be the major viral antigen that elicits protective antibodies [5,6]. Therefore, we chose to use a recombinant form of this antigen, namely, rE2 of EEEV and WEEV, to immunize mice and generate NAbs. A total of 73 MAbs, 7 reactive to EEEV V105-00210 rE2 and 66 reactive to WEEV Fleming rE2 in ELISA, were generated. None of EEEV V105-00210 reactive MAbs bound recombinant or authentic E2 in denaturing Western blotting, whereas 55 out of the 66 WEEV Fleming reactive MAbs bound to rE2 of WEEV Fleming in denaturing Western blotting. Two of these 55 anti-WEEV Fleming rE2 clones, namely, 8C2 and 8H10, were found to have neutralizing activity against WEEV Fleming infectivity for Vero cells. However, none of the seven anti-EEEV MAbs were able to neutralize EEEV PE6 infectivity for Vero cells, although it is possible that these clones might neutralize the homologous strain, namely, EEEV V105-00210, which was not tested in this study.

The host antibody response plays a pivotal role in the prevention of, recovery from, and treatment of viral infections. Antibodies against the numerous epitopes on virus proteins can be divided into neutralizing and non-neutralizing categories. Only a small fraction of antibodies are NAbs, which are capable of blocking the virus infection of host cells by interfering with virion binding to receptors on cells, blocking uptake, preventing uncoating of the genomes in cells, or inducing aggregation of virus particles [39]. The vast majority of antibodies are non-NAbs, which bind specifically to virus particles, but do not neutralize viral infectivity [40]. This may explain why only 2 out of 14 anti-WEEV MAbs that were tested had neutralizing activity. However, other factors, such as improperly folded rE2 and inadequately presented rE2 as compared with the natural E1–E2 dimers, are not excluded as explanations. 

The E2 protein of alphaviruses binds to host cell surface receptors to trigger virus internalization and transport the virus into acidic intracellular vesicles. Within the low pH environment of the endosome, conformational changes in the envelope proteins allow E1 to insert its hydrophobic fusion loop into the membrane of the endosome. Additional conformational changes bring the endosomal and viral membranes together, thus causing membrane fusion and virus infection [41]. E1 plays a pivotal role in alphavirus internalization into host cells. Das’ study showed that both E1 and E2 were directly involved in contact with the host immune system and elicitation of host immune responses [42]. Indeed, E1 and E2 are translated and processed through the endoplasmic reticulum and Golgi apparatus together and E2 would not be expressed on the surface of infected cells without association with E1. Without E1, E2 might not be processed and folded into the same conformation that it has on the surface of the virus [43]. Therefore, our second approach was to use both E1 and E2 as immunogens in either heterodimer or mixture formats from various strains to immunize the mice and increase the probability of eliciting not only neutralizing but also cross-strain neutralizing anti-EEEV or anti-WEEV hybridoma clones. As such, the inactivated EEEV and WEEV viruses that were provided by the Zoetis vaccine were used to prime the mice. The EEEV and WEEV strains in this vaccine are unknown due to commercial confidentiality. Afterward, the mice were given two boosters immunizations, providing all the structural proteins of EEEV PE6 (adenovirus vector) or WEEV 71V-1658 (DNA plasmid vector, supplemented with rE1 and rE2 proteins of WEEV Fleming). In this way, the opportunities for B-cell clones to recognize epitopes that were shared by different EEEV or WEEV strains were improved. 

In general, the conventional approach for isolating NAbs from immunized mice is to identify antigen-binding clones first using an immunoassay and then identify neutralizing clones via an in vitro neutralization assay using these antigen-binding clones. Since infectious alphaviruses are CL3 agents, which cause potentially lethal human diseases, their use in an immunoassay is prohibited in CL2 laboratories. Instead, either inactivated viruses or recombinant antigens are used for the immunoassay. However, the conformation of inactivated virus or recombinant antigens might not be identical to an authentic virus. Any change might affect the neutralizing epitopes that are recognized by NAbs and these NAb clones would be missed by the immunoassay. In order to overcome this hurdle, an in vitro neutralization assay using live viruses was applied to screen for anti-EEEV or anti-WEEV neutralizing clones from approximately 5000 hybridoma clones each. This led to the identification of three anti-EEEV and five additional anti-WEEV NAbs.

Eight WEEV strains were previously compared based on virulence and genetic diversity, where the eight strains could be divided into two groups: a high-virulence group consisting of strains California, Fleming, and McMillan; and a low-virulence group, including strains CBA-87, Mn548, B11, Mn520, and 71V-1658 [32]. To investigate whether these NAbs had cross-strain activity against EEEV or WEEV infectivity, the most promising candidates with the highest neutralizing titers (two anti-EEEV NAbs and two anti-WEEV NAbs) were evaluated against multiple EEEV or WEEV strains, respectively. Anti-EEEV NAbs G1-2-H4 and G1-4-C3, which were developed from the second immunization approach, were able to neutralize all EEEV strains tested and one Madariaga strain tested.

Meanwhile, anti-WEEV NAbs neutralized six out of eight tested strains. Only the California and McMillan strains were resistant to neutralization by these two NAbs. It is notable that NAbs G8-2-H9 and 12WA had activity against members of both groups. Interestingly, the California and McMillan strains share four amino acids in E2 that are not present in the other strains of WEEV tested [32], suggesting the epitope for these two NAbs may involve this region. WEEV 71V-1658 was used to initially screen the hybridoma clones, and it is possible to have missed antibodies that neutralize the California and McMillan strains via an epitope involving this four-amino-acid stretch. Both G8-2-H9 and 12WA were developed using the second immunization approach in which the mice were immunized with the antigens from multiple WEEV strains. Unlike G8-2-H9 and 12WA, 8C2 was developed from the first immunization approach in which the mice were immunized with the rE2 from only the Fleming strain. This difference in the immunization approaches may explain why 8C2 was the only identified anti-WEEV NAb that did not neutralize any of the heterologous strains tested. These results indicate that these cross-strain NAbs recognize a common or conserved neutralizing epitope that is shared by several strains of EEEV or WEEV, respectively. Furthermore, the results indicated that immunization strategies for antiviral MAbs should include antigens from multiple strains to improve the potential for generating MAb-based MCMs with activity across the target species.

In this study, the five most promising NAbs were evaluated in an in vivo prophylactic efficacy setting, inoculating mice one day prior to aerosol exposure to the virus. All five NAbs demonstrated high levels of protection against EEEV or WEEV infection in mice. Regardless of the NAb dose, complete protection was afforded by G1-4-C3 against a lethal aerosol exposure of EEEV PE6, where surviving animals were free of clinical signs throughout the study and maintained typical weight profiles. Complete protection was also afforded by 8C2 at both NAb dose levels against a sub-lethal aerosol exposure of WEEV Fleming, where surviving animals were free of clinical signs throughout the study and maintained typical weight profiles and clinical scores. The lower clinical score in the WEEV control animals relative to EEEV control animals was reflected by the less than 100% mortality in the WEEV control mice. This was due to the nature of aerosol challenge experiments, where the anticipated, or intended, dose and the actual delivered dose might vary. Importantly, we were able to calculate the actual delivered dose after exposure and it was thus clear that the delivered dose of WEEV was less than intended and sub-lethal for this established model [37], whereas the EEEV challenge was in the intended lethal range. Nevertheless, the extensive clinical score data, and its correlation with the level of mortality, provided a clear test of the ability of the NAbs to protect against a WEEV challenge.

To date, this is the first description of cross-strain neutralizing anti-EEEV and anti-WEEV NAbs. These data provide an evidence base on which to further explore the potential of NAbs as cross-strain antibody-based MCMs against these encephalitic alphaviruses, for which there are no licensed MCMs for use in humans.

## Figures and Tables

**Table 1:** Anti-EEEV NAbs.

Neutralizing Titers (µg/mL)				
NAbs	PE6	FL93-969 435731	Williams	Madariaga Virus
G1-2-H4	2.4	+	+	+
G1-4-C3	7.2	+	+	+
G1-4-A2	21.6	N/A	N/A	N/A

N/A, not tested; +, 100 µg/mL antibody resulted in at least 50% inhibition of CPE caused by 100 TCID_50_.

**Table 2:** Anti-WEEV NAbs.

Neutralizing Titers (µg/mL)								
NAbs	Fleming	71V-1658	CBA-87	B11	Mn548	Mn520	California	McMillan
G8-2H9	0.03	+	+	+	+	+	-	-
12WA	0.03	+	+	+	+	+	-	-
8C2	0.03	-	-	-	-	-	-	-
8H10	18.00	N/A	N/A	N/A	N/A	N/A	N/A	N/A
G4-2-A4	0.90	N/A	N/A	N/A	N/A	N/A	N/A	N/A
G4-2-H1	0.27	N/A	N/A	N/A	N/A	N/A	N/A	N/A
G4-2-H7	0.27	N/A	N/A	N/A	N/A	N/A	N/A	N/A

N/A, not tested; +, 100 µg/mL antibody resulted in at least 50% inhibition of CPE caused by 100 TCID_50_; -, 100 µg/mL antibody did not result in any inhibition of CPE caused by 100 TCID_50_.

## Data Availability

The data presented in this study are contained within the article.
